# Supplementary material for: The causal relationship between the human gut microbiota and pyogenic arthritis: a Mendelian randomization study
Source: Front Cell Infect Microbiol. 2024 Nov 26;14:1452480. doi: 10.3389/fcimb.2024.1452480 (PMC11629706; doi:10.3389/fcimb.2024.1452480)
Supplement: Supplementary file 1 [file DataSheet1.zip › mendelian test/ebi-a-GCST90027499.csv_leave_one_out.pdf]

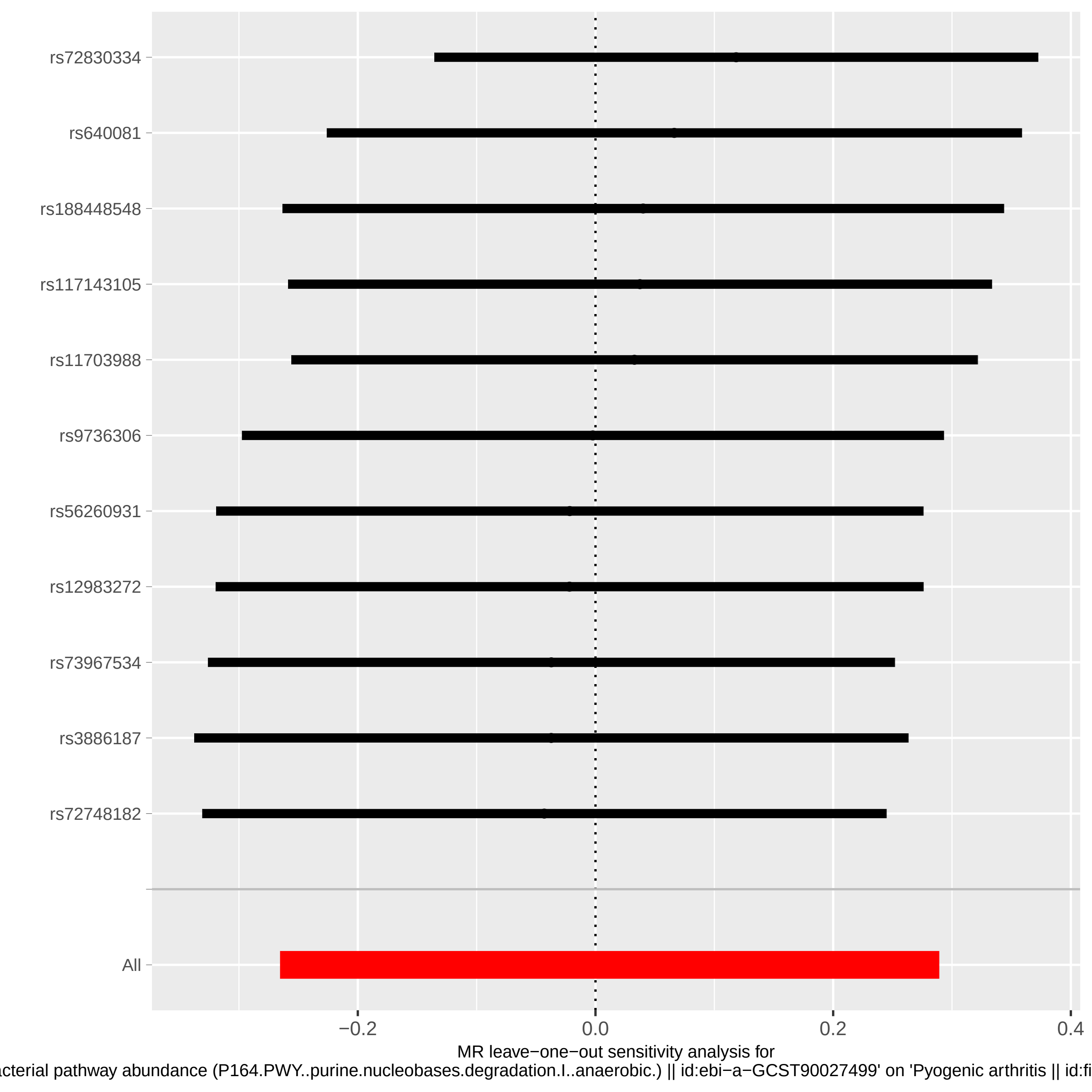
rs72830334
rs640081
rs188448548
rs117143105
rs11703988
rs9736306
rs56260931
rs12983272
rs73967534
rs3886187
rs72748182
All
−0.2
0.0
0.2
0.4
MR leave−one−out sensitivity analysis for
acterial pathway abundance (P164.PWY..purine.nucleobases.degradation.I..anaerobic.) || id:ebi−a−GCST90027499' on 'Pyogenic arthritis || id:fi
